# Standard comparison of local mental health care systems in eight European countries

**DOI:** 10.1017/S2045796017000415

**Published:** 2017-09-18

**Authors:** M. R. Gutiérrez-Colosía, L. Salvador-Carulla, J. A. Salinas-Pérez, C. R. García-Alonso, J. Cid, D. Salazzari, I. Montagni, F. Tedeschi, G. Cetrano, K. Chevreul, J. Kalseth, G. Hagmair, C. Straßmayr, A. L. Park, R. Sfectu, T. Ala-Nikkola, J. L. González-Caballero, L. Rabbi, B. Kalseth, F. Amaddeo, For the REFINEMENT Group

**Affiliations:** 1 PSICOST Research Association, Departamento de Psicología, Universidad Loyola Andalucía, Sevilla, Spain; 2 Centre for Mental Health Research, Research School of Population Health College of Medicine, Biology and Environment, Australian National University, Research School of Population Health, 63 Eggleston Road, Acton, ACT 2501, Australia; 3 Departamento de Métodos Cuantitativos, Universidad Loyola Andalucía, Sevilla, Spain; 4 Mental Health & Addiction Research Group, IDIBGI-Institut d'Assistència Sanitària, Girona, Spain; 5 Section of Psychiatry, Department of Neurological, Biomedical and Movement Sciences, University of Verona, Italy; 6 Univ. Bordeaux, Inserm, Bordeaux Population Health Research Center UMR1219, Team HEALTHY, F-33000 Bordeaux, France; 7 Social Care Workforce Research Unit, King's Policy Institute, King's College London, London, UK; 8 Université Paris Diderot, Sorbonne, Paris, France; 9 Inserm, ECEVE, U1123, F-75 010 (Paris, France); AP-HP, URC-Eco, Paris, France; 10 Department of Health Research, SINTEF Technology and Society, Trondheim, Norway; 11 IMEHPS.Research, Vienna, Austria; 12 Personal Social Services Research Unit, LSE Health and Social Care, London School of Economics and Political Science London, UK; 13 Institute for Economic Forecasting, Bucharest, Romania; 14 Helsinki University and Department of Mental Health, National Institute for Health and Welfare (THL), Helsinki, Finland; 15 Department of Statistics and Operations Research, University of Cadiz, Spain

**Keywords:** Community-balanced care, main type of care, mental health care comparison, Mental Health System

## Abstract

**Aims.:**

There is a need of more quantitative standardised data to compare local Mental Health Systems (MHSs) across international jurisdictions. Problems related to terminological variability and commensurability in the evaluation of services hamper like-with-like comparisons and hinder the development of work in this area. This study was aimed to provide standard assessment and comparison of MHS in selected local areas in Europe, contributing to a better understanding of MHS and related allocation of resources at local level and to lessen the scarcity in standard service comparison in Europe. This study is part of the Seventh Framework programme REFINEMENT (Research on Financing Systems’ Effect on the Quality of Mental Health Care in Europe) project.

**Methods.:**

A total of eight study areas from European countries with different systems of care (Austria, England, Finland, France, Italy, Norway, Romania, Spain) were analysed using a standard open-access classification system (Description and Evaluation of Services for Long Term Care in Europe, DESDE-LTC). All publicly funded services universally accessible to adults (≥18 years) with a psychiatric disorder were coded. Care availability, diversity and capacity were compared across these eight local MHS.

**Results.:**

The comparison of MHS revealed more community-oriented delivery systems in the areas of England (Hampshire) and Southern European countries (Verona – Italy and Girona – Spain). Community-oriented systems with a higher proportion of hospital care were identified in Austria (Industrieviertel) and Scandinavian countries (Sør-Trøndelag in Norway and Helsinki-Uusimaa in Finland), while Loiret (France) was considered as a predominantly hospital-based system. The MHS in Suceava (Romania) was still in transition to community care.

**Conclusions.:**

There is a significant variation in care availability and capacity across MHS of local areas in Europe. This information is relevant for understanding the process of implementation of community-oriented mental health care in local areas. Standard comparison of care provision in local areas is important for context analysis and policy planning.

## Introduction

Mental disorders are among the most common and disabling health conditions worldwide and should, therefore, be considered as a top global health priority. Every year over a third of the total EU population suffers from mental disorders, which are the largest contributor to the morbidity burden in these world regions (Wittchen *et al*. 2011). However, there is an important gap between such burden and the availability of resources to manage and reduce it (Patel *et al*. 2013). To satisfactorily bridge this gap, more information is needed about existing infrastructures for adults across Europe at national and local levels. This information is also important with regard to budget allocation and control of expenditure, as well as for efficiency analysis and policy planning (WHO, 2013).

The World Health Organization (WHO) Mental Health Atlas series (WHO, 2011) reported that Mental Health Systems (MHSs) are constantly subjected to change and are being reviewed and redesigned (WHO, 2013). These changes reflect, in part, the growing evidence of what constitutes cost-effective and appropriate care. Many systems now aim to establish mental health services that are local and community-based, organised around the needs of the population in specific catchment areas (Thornicroft & Tansella, 2014) and based on the development of alternative community and recovery-oriented services (Becker & Vázquez-Barquero, 2001).

To monitor, evaluate and communicate the extent to which various aspects of the health system meet key objectives, many countries in Europe are moving towards elaborate systems of health care performance assessment (Smith, 2009). Relevant initiatives in this area include the Quality Indicator for Rehabilitative Care (QuIRC), a measure for the assessment of the quality of care provided in psychiatric and social care facilities; as well as the MENDiT, a tool that provides an objective assessment of a country's level of deinstitutionalisation. Both created within the European project ‘Development of a Measure of Best Practice for People with Long Term Mental Illness in Institutional Care’ (DEMOBinC) (Killaspy *et al*. 2016*a*, *b*; Taylor Salisbury *et al*. 2016). Another initiative is the study carried out by the network of mental health experts to analyse the level of implementation at a system level of Quality Monitoring Programmes for Mental HealthCare (QMP-MHC) (Bramesfeld *et al*. 2016).

Regarding service comparison, instruments like the European Service Mapping Schedule (ESMS) (Johnson *et al*. 2000) developed by the EPCAT group (European Pyschiatric Care Assessment Team), and later adaptations (i.e. Description and Evaluation of Services for Disabilities in Europe (DESDE) (Salvador-Carulla *et al*. 2006) and Description and Evaluation of Services for Long Term Care in Europe (DESDE-LTC) (Salvador-Carulla *et al*. 2013)) provide a standardised description of services in local areas, to identify patterns of care and gaps in service availability. These instruments have been used within different international studies like the European Psychiatric Services: Inputs linked to Outcome Domains and Needs (EPSILON) that identified and described mental health services for people with schizophrenia in five catchment areas (Becker *et al*. 2002). It has also been used for the comparison of small catchment areas in Italy and Spain (Salvador-Carulla *et al*. 2005); for the regional analysis of service delivery and social and demographic factors in Piedmont (Italy) (Tibaldi *et al*. 2005); for the comparison of regions in Norway and Russia (Barent) (Rezvyy *et al*. 2007); and in national comparisons in Finland, Germany, Poland and Catalonia (Spain) (Böcker *et al*. 2001; Trypka *et al*., 2002; Ala-Nikkola *et al*. 2014*a*; Fernandez *et al*., 2015).

However, data on MHS comparison across Europe is scarce, mainly due to two fundamental problems encountered when comparing services: (a) terminological variability: the names of the services do not always reflect the activity they perform, and (b) a commensurability problem: under the umbrella term ‘service’ there are different units of analysis (e.g. main types of care, care modalities, care programmes, care packages, single interventions, activities, etc.); these problems hamper like-with-like comparisons.

This study was part of the REFINEMENT project (Research on Financing Systems’ Effect on the Quality of Mental Health Care in Europe) (http://www.refinementproject.eu/), which was funded by the European Seventh Framework Programme (FP7) and implemented in order to better understand the patterns of mental health care provision, and the balance between hospital and community care (e.g. between residential and day care) in Europe. This specific study was aimed at:

1. Assessing and describing the availability, diversity and capacity of mental health care resources in selected study areas of eight European countries (Austria, England, Finland, France, Italy, Norway, Romania and Spain); enabling international comparisons across jurisdictions.
2. Contributing to overcome the scarcity in standard service comparison in Europe, by quantifying and objectifying the variability in the provision of mental health care in study areas of different countries in Europe. This achievement will allow a better understanding of MHS and information for the allocation of resources at the local level.

## Method

This study was jointly coordinated by PSICOST, Loyola University (Spain) and the University of Verona (Italy) within the REFINEMENT project. A formal partnership with official agencies in two of the eight participating countries was established with the Mental Health Unit at the Department of Health in Catalonia (Spain), and with the Department of Health in Finland. This study is part of the REMAST work package (Refinement Mapping Services Tool) of the REFINEMENT project for developing a combined set of tools that may facilitate the monitoring, reviewing and improvement of MHS in eight study areas. The full description of the method followed in this study has been previously published and is available online (Salvador-Carulla *et al*. 2015). It mainly uses a classification of services questionnaire to evaluate provision of mental health care, the DESDE-LTC tool (Description and Evaluation of Services and Directories in Europe for Long Term) (Salvador-Carulla *et al*. 2000, 2011*a*, *b*, 2013; Poole, 2004; Johnson *et al*. 2011).

### Catchment areas

Eight catchment study areas were selected in the eight countries according to the following inclusion criteria: (i) a population size between 200 000 and 1 500 000 inhabitants; and (ii) coverage of at least one health district, which was not limited to a macro-urban area within a municipality; (iii) availability of reliable sources of information on the local MHS. The following areas were selected (for readability purposes a short name is listed in brackets): Industrieviertel in the Province of Lower Austria (Industrieviertel); Hampshire including Portsmouth and Southampton Unitary Authorities in England (Hampshire); Helsinki and Uusimaa Hospital District in Finland (Helsinki and Uusimaa); the public health-oriented hospital of Loiret in France, ‘Georges Daumézon Hospital Center’ (Loiret); ULSS20-Verona Mental Health Department in Veneto, Italy (Verona); Sør-Trøndelag in Norway (Sør-Trøndelag); Jud-Suceava in Romania (Suceava) and Girona Health District in Catalonia, Spain (Girona). Data collected in the eight REFINEMENT study areas refer to years 2008–2011. Data were collected from the most recent year when not available for this period.

### Material

REMAST is comprised of a battery of checklists/inventories, instruments and indexes for mental health service and system assessment. It contains five main sections: (A) Population Data; (B) Verona SES (Socio-economic Status) Index (Tello *et al*. 2005); (C) Mental Health System Checklist describing policies and organisation of mental health care through selected WHO-AIMS 2.2 items (WHO, 2005); and (D) the Mental Health Services Inventory, allowing the mental health services of a selected study area to be classified according to the DESDE-LTC instrument, for providing detailed descriptions of the coding and characteristics of services from all relevant sectors. The indicator set of sections (A) and (B) was built using the European Sociodemographic Schedule (ESDS) (Beecham & Johnson, 2000) and the DESDE-LTC inventory (PSICOST Mental Health indicators set) (Salvador-Carulla *et al*. 2010).

Country comparisons across local areas (meso-level) were based on the assessment of two ‘units of analysis’ described in the DESDE-LTC: (1) Basic Stable Input of Care (BSIC), and (1) Main Type of Care (MTC). BSICs are the minimal stable units of production of care identified in a service with temporal and organisational stability, defined as a stable team of professionals that provide coordinated care to a discrete target group of health consumers. Its operational description is based on its main characteristics of service provision (placement, users, staff and management). MTC is the descriptor of the basic activity carried out in one BSIC. The DESDE-LTC comprises 90 MTCs or codes for the classification of BSICs. Note that when referring to placement capacity of a service, ‘places’ is the general term used for addressing both beds in residential settings and places in day care settings.

### Sample

Services with public coverage and universal access, providing health and social care to adults aged ≥18 years with a psychiatric disorder (F2–F6 10th revision of the International Statistical Classification of Diseases and Related Health Problems (ICD-10) diagnostic (World Health Organization, 2010)) available during the year 2010 were gathered in an *ad hoc* document. Services not intended exclusively for mental health care users were excluded from the study. After completing the database in August 2012, various reviews and updates followed to create the final dataset in July 2013 with 857 BSICs and 1018 MTCs.

### Procedure

In this study, we describe direct provision of care in three main coding branches of DESDE-LTC (refer to supporting Table 4 for a description of codes): Outpatient or ambulatory care (O), Day care (D) and Residential care (R). MTCs with codes referring to similar activities and provided in similar settings (either HOSPITAL or COMMUNITY based) were analysed together (e.g. DESDE-LTC codes R4-R6 for services providing non-acute care in hospital settings), providing a final reduced number of 18 aggregated codes which are represented in tables and figures. The disaggregated information of full service provision in the study areas is available in the REFINEMENT Atlas on the website. Romania is represented with a dashed line in figures to note that mental health care is not organised by reference catchment areas or sectors and cannot be fully compared (like-with-like comparison) with the rest of areas described in this study.

The study is focused on the availability of MTCs in eight local study areas, the span of diversity of services available (measured as the diversity of MTC codes available in each area), and the placement capacity (beds and day care places per 100 000 inhabitants). In addition, sociodemographic data of the assessed areas were collected using the sections A (Population Data) and B (Verona SES Index) of the REMAST tool.

## Results

### Sociodemographic indicators studied in selected areas of eight European countries

The main sociodemographic indicators in the study areas are shown in Table 1. Industrieviertel rated the highest values for divorced or widowed persons in comparison with other areas; the low unemployment rate was also significant. Hampshire showed one of the highest population densities; the unemployment rate in relation to this figure was quite low; the ageing index showed evidence of one of the most aged populations assessed in the study. In Helsinki and Uusimaa, the low population density was noteworthy in relation to a broader land area, the highest rates of one-person household evaluated in the study, and to the fact that the average household in the area was of only two persons. Unemployment and dependency rates were low in this area. Loiret showed the second highest dependency ratio of the study (rate of individuals below 15 or over 65 years of age) and low rates for immigration. Verona registered the highest ageing index, and very high levels of population density and immigration; census data responded mainly to the year 2001. Sør-Trøndelag was the largest area with the lowest population density. The unemployment index in this area was also the lowest found in the study. Like in Finland, a high share of one-person households was also reported. Suceava registered the highest dependency index and highest number of people per household. It also showed an important rate of unemployment and the lowest rates for ageing and immigration; these values responded mainly to the reference year 2002. Girona showed the highest unemployment and immigration rates, and the lowest number of divorced and widowed people. The rate for one-person household was also low in this area.

**Table 1.** Sociodemographic characteristics in eight study areas

	Study area sociodemographics							
Indicator	Industrieviertel (Austria)	Hampshire (England)	Helsinki Uusimaa (Finland)	Loiret (France)	Verona (Italy)	Sør-Trøndelag (Norway)	Suceava (Romania)	Girona (Spain)
Population (>18 years)	445 748 (2011)	1 364 799 (2010)	1 206 446 (2010)	422 853 (2008)	393 402 (2010)	225 081 (2010)	484 212 (2002)	599 473 (2010)
Land area (km^2^)	3921	3769	8751	5626	1061	18 856	8553	5585
Population density (inhb./km^2^)	136.79 (2001)	459.45 (2010)	176.56 (2011–12)	97.36 (2008)	416.85 (2001)	15.60 (2011)	80.49 (2002)	132.61 (2010)
Ageing index (>65/<15 × 100)	95.39 (2001)	100.66 (2011)	82.17 (2010)	87.92 (2008)	144.10 (2010)	81.42 (2012)	65.06 (2002)	98.29 (2010)
Dependency ratio (<15 and >65/15–4 × 100)	47.28 (2001)	52.43 (2011)	44.82 (2010)	55.56 (2008)	53.51 (2010)	49.55 (2012)	55.60 (2002)	46.20 (2010)
% of one person household	32.66 (2001)	27.73 (2001)	41.37 (2011)	32.03 (2008)	29.16 (2001)	40.78 (2011)	17.30 (2002)	17.94 (2007)
Average n/p household	2.33 (2001)	2.37 (2011)	2.07 (2011)	2.36 (2008)	2.44 (2001)	2.21 (2011)	3.10 (2002)	2.62 (2007)
% of immigrants	9.39 (2011)	20.95 (2011)	6.14 (2011)	5.91 (2008)	12.24 (2010)	6.64 (2012)	0.016 (2006)	21.60 (2010)
% of unemployment	4.60 (2008)	5.8 (2011)	7.35 (2010)	8.5 (2012)	4.21 (2001)	2.79 (2010)	12.70 (2002)	18.28 (2010)
% of divorced/widowed	14.86 (2001)	13.21 (2001)	14.39 (2009)	11.37 (2008)	9.79 (2010)	11.73 (2011)	11.01 (2002)	7.45 (2007)

Source: REFINEMENT countries.

### Availability of MTCs and BSICs capacity according to DESDE-LTC

A total of 857 teams or BSICs were identified and coded in 1018 MTCs according to the DESDE-LTC system (Table 2). The rate of BSICs per 100 000 inhabitants (care availability), the number and description of different MTCs (diversity), and the rate of beds and places assigned to persons experiencing mental disorders (placement capacity) were described for each study area. In general terms, Helsinki and Uusimaa together with Hampshire showed the highest diversity of types of care, whereas Suceava and Loiret were the least diverse (Table 2).

**Table 2.** Availability, placement capacity and diversity of units of analysis (BSIC/MTC) and typologies of DESDE-LTC codes in eight study areas

Description of the units of analysis in the study areas									
Indicator		Industrieviertel (Austria)	Hampshire (England)	Helsinki Uusima (Finland)	Loiret (France)	Verona (Italy)	Sør-Trøndelag (Norway)	Suceava (Romania)	Girona (Spain)
General provision of BSIC/MTC									
Availability of BSIC	Gross number	148	82	238	174	46	102	28	39
	Rate per 100 k inhabitants	33.20	6.01	19.72	41.15	11.69	45.32	5.78	6.51
Availability of MTC	Gross number	156	108	256	202	71	149	36	40
	Rate per 100 k inhabitants	35.00	7.91	21.22	47.77	18.05	66.20	7.43	6.67
Placement capacity	Gross number	669	562	3.901	431	480	258	1.261	724
	Rate per 100 k inhabitants	150.08	41.18	323.35	101.93*	122.01	114.63*	260.42	120.77
Diversity (different MTCs)		17	24	29	7	13	19	7	14
Typologies of DESDE-LTC codes in eight study areas									
Indicator		Industrieviertel (Austria)	Hampshire (England)	Helsinki Uusima (Finland)	Loiret (France)	Verona (Italy)	Sør-Trøndelag (Norway)	Suceava (Romania)	Girona (Spain)
Aggregated typology of MTC (DESDE-LTC codes)		Availability + MTC (DESDE-LTC code)							
Acute care in hospitals	R1, R2, R3.0	2-R2	6-R1, 11-R2	2-R1, 20-R2	9-R2	4-R2	3-R2	4-R2	1-R2
Acute care in the community (non-hospital)	R0, R3.1.1						1R3.1.1		
Non-acute care in hospitals	R4, R6		3-R4, 1-R6	14-R4, 23-R6		2-R4	10-R4, 1-R6	1-R6	1-R4, 1-R6
Non-acute care in the community (alternatives to hospitalisation)	R5, R7		4-R5	2-R5, 5-R7		6-R5			
Residential care in the community, high intensity	R8, R11	6-R11		48-R11			2-R8		1-R11
Residential care in the community, other intensity	R9, R10, R12, R13, R14	1-R9, 3-R10, 1-R12, 1-R13	3-R9, 1-R10	1-R9, 34-R12, 2-R13	15-R9	11-R9, 10-R10, 1-R14		8-R12	10-R13
Acute day care	D1.1, D1.2, D10	2-D1.1		8-D1.2	15-D1.2	4-D1.1		2-D10	1-D1.2
Non-acute, health-related day care (eg. rehabilitation centres with health professionals)	D4.1, D8.1		1-D8.1	9-D4.1, 2-D8.1	5-D4.1	8-D4.1	1-D4.1		3-D4.1
Work-related day care	D2, D3, D6, D7	1-D2, 5-D3	1-D2, 1-D3, 4-D7	2-D2, 14-D3, 3-D7			2-D3, 2-D7		6-D2, 2-D3
Other types of day care (social, education, etc.)	D4.2, D4.3, D4.4, D8.2, D8.3, D8.4, D5, D9	6-D4.3, 1-D8.3, 1-D5	3-D4.3, 1-D4.4, 5-D8.3	1-D4.2, 3-D5,			3-D4.3, 19-D8.3		5-D4.3
Acute outpatient care, health-related, non-mobile	O3.1, O4.1	2-O3.1		2-O3.1, 9-O4.1	2-O3.1	3-O3.1	1-O3.1	2-O3.1	1-O3.1
Acute outpatient care, non-health-related, non-mobile	O3.2, O4. 2			3-O4.2					
Acute outpatient care, health-related, mobile (e.g. crisis teams)	O1.1, O2.1		5-O1.1, 11-O2.1	1-O2.1		1-O1.1, 3O2.1	2-O2.1		
Acute outpatient care, non-health-related, mobile (e.g. social outreach teams)	O1.2, O2.2						1-O2.2		
Non-acute outpatient care health-related, non-mobile (e.g. community mental health centres)	O8.1, O9.1, O10.1	17-O8.1, 99-O9.1	1-O8.1, 1-O9.1, 1-O10.1	3-O8.1, 33-O9.1, 3-O10.1	154-O9.1	14-O9.1	28-O8.1, 41-O9.1, 1-O10.1	18-O8.1 1-O10.1	4-O8.1, 3-O9.1
Non-acute outpatient care non-health-related, non-mobile	O8.2, O9.2, O10.2	1-O8.2 7-O9.2		1-O10.2					
Non-acute outpatient care health-related, mobile	O5.1, O6.1, O7.1		2-O5.1, 34-O6.1, 2-O7.1	6-O6.1, 1-O7.1	2-O6.1	4-O5.1	22-O5.1, 3-O6.1		1-O5.1
Non-acute outpatient care non-health-related, mobile	O5.2, O6.2, O7.2		3-O5.2, 3-O7.2	1-O5.2			6-O5.2		

Source: DESDE-LTC, REFINEMENT countries. In brackets: raw data.

* Lack information on day place.

#### Residential care availability and capacity (Fig. 1)

**Fig. 1.**
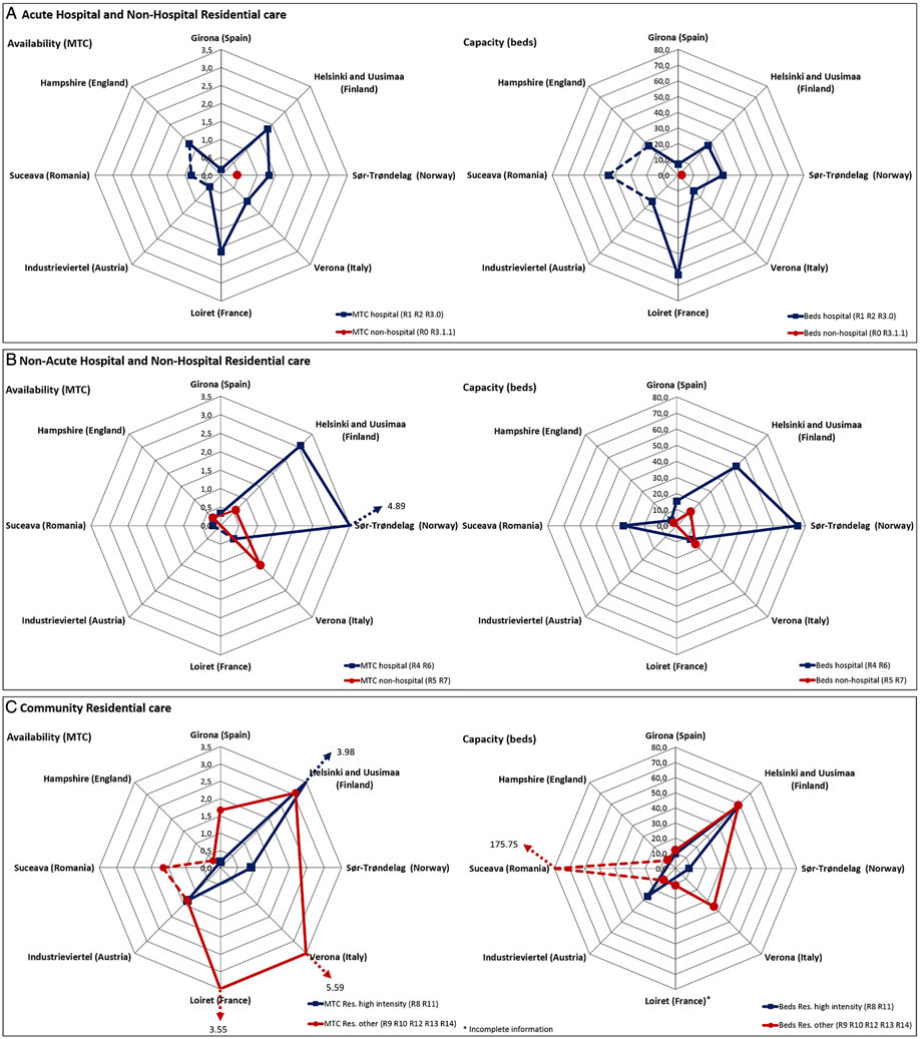
Acute, non-acute and community residential care per 100 000 inhabitants.

In the areas of Loiret and Helsinki and Uusimaa, the rate of services found for the provision of acute care within hospital settings was much higher compared with the other countries of the study (R2), whereas in Girona the availability was very low with less than one service per 100 000 inhabitants (Fig. 1*a*). Sør-Trøndelag was the only area showing this type of acute care but in off-hospital facilities (R3.1.1); the high rates of services and places identified in Sør-Trøndelag for non-acute hospital care (R4) is also noteworthy in comparison with other areas. This typology of care was present neither in Industrieviertel nor in Loiret. Alternatives to hospitalisation such as non-acute residential care with 24 h physician cover in the community (R5, R7) were mainly present in Verona and, to a lesser extent, in Hampshire and Helsinki and Uusimaa (Fig. 1*b*). In this area, the high availability of residential facilities in the community with 24 h support (R11) was particularly relevant (Fig. 1*c*). Other typologies of residential care were significant in Verona, Helsinki and Uusimaa and Loiret for different ranges of stay and support (R9, R10 and R12) and in Suceava, which showed extremely high rates of institutional beds (R12). It is important to note that a significant number of residential services in the community are not being specifically designed for mental health. This is particularly significant in the area of Sør-Trøndelag where there are more than 50 places per 100 000 inhabitants (18+) in supported housing (flats) that were not included in the study since they were not exclusively designed for mental health users.

#### Day care availability and capacity (Fig. 2)

**Fig. 2.**
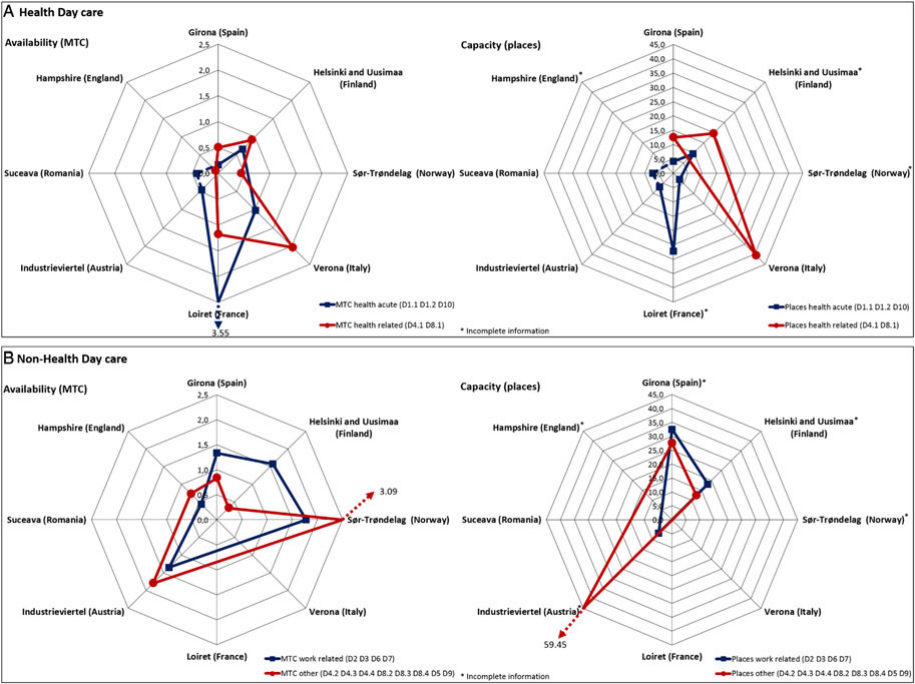
Health- and non-health-related day care per 100 000 inhabitants.

Loiret showed the highest rates of acute care services and places in day hospitals (D1.1 and D1.2). As for non-acute day care (D4.1 and D8.1), all areas presented quite similar rates except for Suceava and Industrieviertel, which did not provide this typology of care. Verona registered the largest number of places, while this information was not available for Sør-Trøndelag, Hampshire or Loiret. Sør-Trøndelag and Loiret lack information on places as they use an average occupancy indicator instead of the actual number of users. This is, nevertheless, typically found in non-structured facilities (D5). Industrieviertel showed the highest rates of places for social day care (D4.3). Social clubs are typically accessible for a large number of users at the same time, which results in higher rates of places than MTCs. In Suceava, hospitals offered generic and unspecific day care that included mixed acute and non-acute care delivery in observation wards not seen elsewhere that were coded as ‘other day care’ (D10). For clustering purposes, this particular service was grouped in acute care, although it provided other types of care (Fig. 2*a*). Almost all areas, except Loiret, Suceava and Verona, included in their systems other types of day care related to work, social or education activities. Industrieviertel showed the highest rates of places, and Sør-Trøndelag the highest rates of MTCs for social day care (Fig. 2*b*).

#### Outpatient/ambulatory care availability (Fig. 3)

**Fig. 3.**
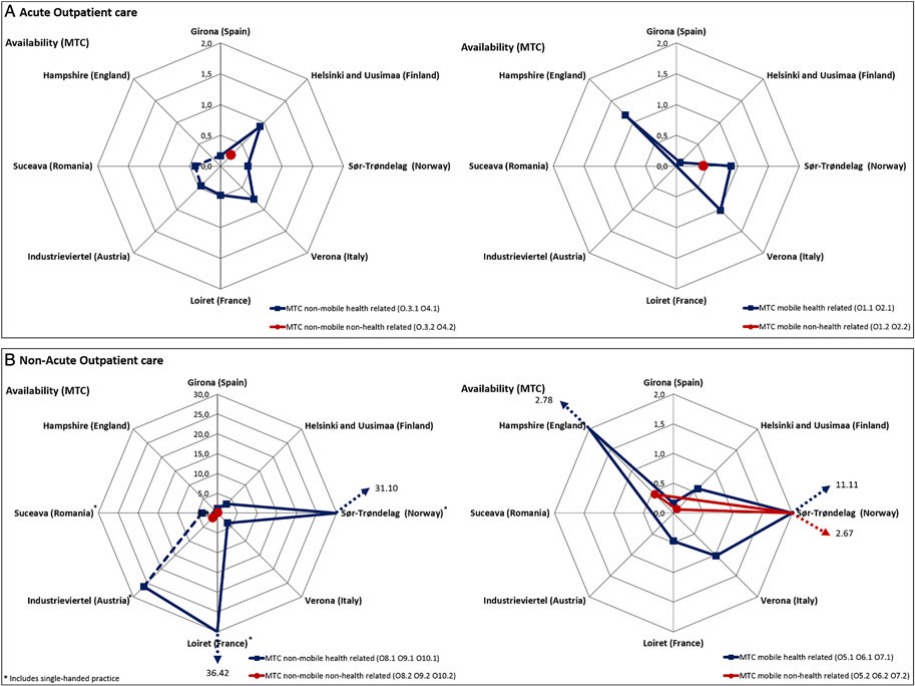
Acute and non-acute outpatient care per 100 000 inhabitants.

Only 50% of the areas had mobile ambulatory care (Hampshire, Verona, Sør-Trøndelag and Helsinki and Uusimaa). Hampshire showed the highest rates of the study (O1.1 and O2.1) (Fig. 3*a*). On the other side, all areas except Hampshire presented non-mobile acute care provided 24 h a day (O3), which is generally linked to acute hospital wards (R2) working as a support unit. Helsinki and Uusimaa was the only area offering this type of care but for a limited number of hours (O4.1) inside and outside hospitals. Finally, non-mobile, non-acute, health-related care (O8-O10) typically found in community mental health services was available in all areas with highest rates in Loiret, Sør-Trøndelag and Industrieviertel (Fig. 3*b*).

## Discussion

Context analysis, including the standard description of local areas, their main social and demographic features together with the care delivery system (availability, diversity and placement capacity) is critical for the comparison and understanding of complex systems and interventions (Wahlbeck, 2011; Bosch-Capblanch *et al*. 2012; Bate *et al*. 2014). The core configuration of assessed study areas differed notably among the eight countries. In this regard, the availability of information on the health system at local and national levels is essential for providing informed evidence for health care planning (Raine *et al*. 2016).

### Hospital *v.* community-oriented residential, day and outpatient care

In Loiret, the ‘sector’ model of care implemented in France after 1960 might explain the high rates of acute hospital services (R2). In general terms, in France, hospital-based care is still of overwhelming importance with a consequential impact in the development of community services, and lack of supported housing for the most severe cases (Chapireau, 2005). There was also no separation of acute and non-acute beds within the hospital, unlike other study areas, contributing to a high acute rate. Therefore, patients usually stay longer in acute settings.

In Industrieviertel and Loiret, in the absence of a dedicated non-acute service/unit with 24 h physician cover, patients were taken care of by other types of services (community residential services R8, R9, R11, etc.). This finding may indicate a gap in residential care for long-term patients in these areas. Verona and, to a lesser extent, Hampshire, included residential health care in the community as an alternative to hospitalisation (R5, R7). These services generally represent a step forward in the deinstitutionalisation process. Although not present in Girona, these alternatives to hospital care are available in other areas of Catalonia (Spain) as it has been described in the Mental Health Atlas of this region (Fernandez *et al*. 2015). The high availability of residences (R9 and R10) found in Verona in comparison with other areas may be linked to the history of the development of residential care in the context of the Italian mental health care reform (de Girolamo *et al*. 2002).

Even though the MHS in Finland has been regarded as a benchmark of community care, the pattern of service provision in Helsinki and Uusimaa was more hospital-based than other local systems in Europe. Residences for intensive care (R11) have been rapidly increasing in the area since a new legislation was introduced in 1990 (Mental Health Act, 1990). In spite of the development of community mental health services in this region, a majority of resources is still allocated to residential care. This may represent a trans-institutionalisation (a shift from hospitals to other institutions) as well as private entrepreneurship. The high rates of dependency and one-person households in comparison with other areas might be related to a higher need of hospital and residential placement capacity.

In Sør-Trøndelag, many of the non-acute hospital services (R4 and R6) provide specialised care for severe mental illness in new health complexes that belong mainly to the district psychiatric centres and are characterised by a higher patient turnover than in traditional medium- and long-term services with a more community-oriented focus (Pedersen & Kolstad, 2009). New types of acute care in hospital precincts were available in Girona.

Mental health in Romania is not organised by catchment areas and is still concentrated in psychiatric hospitals and psychiatric wards of general hospitals, so services in the area of Suceava are available for the whole country. This brought inaccurate comparisons with other country areas, and the adjustment of care availability and capacity per population provided here is just for orientation. Despite the efforts of the psychiatric reform in Romania, Suceava showed a pattern of care characterised by institutionalisation with high rates of beds devoted to long-term residential care in hospital and non-hospital institutions (R6 and R12). A special mention should be made to the identification of a service providing a whole range of typologies of care (from acute to non-acute) in a single BSIC. This particular case highlights the need to combine a standard coding of services with a complementary system to describe the quality of care provided by these BSICs as suggested in the REFINEMENT project (REQUALIT) or the combined use of DESDE-LTC with other international instruments, such as QuIRC (Killaspy *et al*. 2016*b*).

Regarding day care, in Loiret the high rates of acute services and places found may respond to the historical development of the ‘Psychiatrie de Secteur’ (Sectoral Psychiatry) in France in the 1960s and 1970s, particularly in the centre of France where there were many psychiatric hospitals before the reform (Chapireau, 2005). Some of these acute services may be actually functioning as non-acute therapeutic day centres today. Verona showed many small well-structured non-acute services (D4.1) distributed in the area that support care in the community. The same applies to Helsinki and Uusimaa and Girona.

Hampshire was one of the areas with the highest diversity of care, which may be related to the intense transformation of the service delivery system in England, as well as to the high population density and urbanisation of the area (Ala-Nikkola *et al*. 2014*b*, 2016). In comparison with other country areas, there is a low availability of day care. This may be related to a shift from day care services (BSICs) to day programmes or activities provided by outpatient teams (e.g. Community Treatment Teams, Community Mental Health Teams, etc.), thus coded as ‘O’ MTCs. Therefore, Hampshire may be defined by a typology of mobile, acute, ambulatory care (O1 and O2), which is representative of a community-focused model. This care type is also available in Sør-Trøndelag, Verona and Helsinki and Uusimaa, but in these other areas it is not replacing day care. In any case, the shift from day to outpatient outreach care requires a more detailed analysis as it constitutes a major change in the MHS that has not been properly assessed and documented internationally.

Sør-Trøndelag had the highest rates of non-acute outpatient care (mobile and non-mobile). This may be connected to the relatively low population density of this area in comparison to other areas. This included a significant rate of single-handed professionals. It should be noted that in this area much of the ambulatory and day care is provided by the local municipalities as an attempt of contributing to nearness to services in a country with a scattered population settlement (OECD, 2014). In Austria, individual clinics represent the principal type of care although only about a fifth of self-employed psychiatrists have a contract with health insurances. The lack of specific coordination together with the fragmentation of psychiatric services hampers the development of integrated community care (Meise *et al*. 2008).

### Practical implications in the MHS assessed

The findings resulting from the analyses were useful to detect MHS with a stronger community approach such as those in Hampshire, Verona or Girona; areas with a high availability of community, residential and hospital services (areas in Scandinavian countries); and areas where the deinstitutionalisation process is still incomplete, such as Loiret, or at the very early stages of development like in Suceava. The informed evidence obtained from the study caused an important impact in areas such as Helsinki and Uusimaa or Girona where local planners and decision makers activated protocols for incorporating changes in the system and organisation of resources. In Helsinki and Uusimaa, two psychiatric hospitals are planning to provide specialised care with psychiatrists on-site 24 h, and acute residential care for mental disorders is also to be provided in small acute units located at general hospitals, supported by community care teams. In addition, three psychiatric hospitals were closed and patients were reallocated in other settings, offering a range of treatment, care and support tailored to individual needs, rather than simply confining patients. In Girona, the information provided by Remast was key to reactivate the funding for mental health care, reduced by the financial crisis that affected Catalonia and Spain. Despite the weaknesses detected in some resources, Remast helped to strengthen mental health management and contributed to the development of an integrated, community-oriented MHS. In addition, the information provided about the service delivery system in Girona was combined with information about key performance indicators provided by the Requalit tool (evaluation of quality of care) of Refinement. Access to this information facilitated the process through which Girona became a benchmark area in mental health care delivery in Catalonia and Spain. It is important to note that in the areas of Helsinki and Uusimaa and Girona, there was a close cooperation between the local research teams and the public health agencies.

These outcomes represent a solid basis for consistent data harmonisation, collection and benchmarking across European countries.

### Strengths and limitations

The main outcome of the study was to assess and compare the variability of mental health care delivery in selected areas in Europe, being this information a key element to identify and analyse gaps in the care system and to compare them with care needs and demands. Other outcomes were: to identify variation in the care delivery system to analyse waste and quality of care; to increase the knowledge base on service availability and capacity to improve organisation and guide resource allocation; to monitor implementation of service planning and follow-up of local mental health care strategies; to provide standardised information on the local context of care (social and demographic factors and service delivery). In spite of the outcomes, the study faced some limitations. First, there was a high disparity in the data sources and the availability of information across the different local areas. Figures regarding sociodemographic characteristics were collected from the best available local or national source, and should therefore be considered only for orientation. Second, generic services available for the general population (e.g. primary care) or for persons with mental disorders, were not included in this study. Third, these findings are only applicable to the areas under study and cannot be generalised to the whole country. Fourth, this paper does not provide a full description of every area; further information is available on the REFINEMENT Decision Support Toolkit (DST) Appendix (Kalseth *et al*. 2013). The information on service availability, capacity and diversity has to be completed with information on the financing, organisational structures and management and quality of the local MHS. These aspects have been addressed in other sections of the REFINEMENT DST (Kalseth *et al*. 2013). Finally, the classification system, DESDE-LTC, demanded a considerable research effort and high level of training for identification of BSICs and codification of MTCs.

## Supplementary material

For supplementary material accompanying this paper visit https://doi.org/10.1017/S2045796017000415. click here to view supplementary material
