# Supplementary material for: Standard comparison of local mental health care systems in eight European countries
Source: Epidemiol Psychiatr Sci. 2017 Sep 18;28(2):210–23. doi: 10.1017/S2045796017000415 (PMC6998926; doi:10.1017/S2045796017000415)
Supplement: Supplementary file 1 [file S2045796017000415sup001.docx]

**Supporting information**

Table 3. Glossary of terms

| TERM | OPERATIONAL DEFINITION |
| --- | --- |
| Hospital | Hospitals are meso-organisations with a legal recognition in most countries. In those countries where there is no legal basis for deciding what are hospital services and in those cases where doubt exists, services should be classified as hospital if they have more than 20 beds and 24 hours’ resident physician cover. |
| Acute care | Emergency facilities that (i) provide assessment and initial treatment in response to a crisis, deterioration in physical or mental state, behaviour or social functioning which is related to the condition; and (ii) can usually provide a same day response during working hours. At least 20% of the users in the last twelve months do meet the criteria for acute care. |
| Residential care | Facilities that provide beds overnight for users for a purpose related to the clinical and social management of their health condition - users do not make use of such services simply because they are homeless or unable to reach home. |
| Mobile care | In these facilities contact with users occurs in a range of settings including users’ homes, as judged most appropriate by professionals and users. For a service to be classified as ‘home & mobile’, at least 50% of contacts should take place away from the premises at which the service is based. |
| Outpatient care | Facilities that (i) involve contact between staff and users for some purpose related to management of their condition and its associated clinical and social difficulties and (ii) are not provided as a part of delivery of residential or day services. |
| Day care | Facilities that (i) are normally available to several users; (ii) provide some combination of treatment for needs related to MH problems; (iii) have regular opening hours during which they are normally available; and (iv) expect service users to stay at the facilities beyond the periods during which they have face-to-face contact with staff. |
| Health related | Services whose main goal is the specific clinical care and where some of the staff is qualified as health care professionals (Medicine, Nursing, Physiotherapy, Rehabilitation Medicine and Psychology). |
| High intensity | Outpatient care: facilities which have the capacity to make face to face contact with users at least three times per week when clinically indicated for outpatient care.  Day care: admission is usually available within 72 hours.  Residential care: continuous surveillance during 24-hours a day, and/or special isolation measures. |

Source: DESDE-LTC classification system (Salvador-Carulla et al, 2011)

| **Table 4. List of disaggregated MTCs for Residential care (R), Day care (D) and Outpatient care (O) of the DESDE-LTC classification system** | |
| --- | --- |
| **Residential care** | |
| **DESDE-LTC code** | **Description** |
| R0 | Acute, 24 hours physician cover, non-hospital |
| R1 | Acute, 24 hours physician cover, hospital, high intensity |
| R2 | Acute, 24 hours physician cover, hospital, medium intensity |
| R3.0 | Acute, non-24 hours physician cover, hospital |
| R3.1.1 | Acute, non-24 hours physician cover, non-hospital, Health related care |
| R3.1.2 | Acute, non-24 hours physician cover, non-hospital, other care |
| R4 | Non-acute, 24 hours physician cover, hospital, time limited |
| R5 | Non-acute, 24 hours physician cover, non-hospital, time limited |
| R6 | Non-acute, 24 hours psyhician cover, hospital, indefinite stay |
| R7 | Non-acute, 24 hours physician cover, non-hospital, indefinite stay |
| R8.1 | Non-acute, non-24 hours physician cover, time limited, 24-hours support, Less than 4 weeks |
| R8.2 | Non-acute, non-24 hours physician cover, time limited, 24-hours support, Over 4 weeks |
| R9.1 | Non-acute, non-24 hours physician cover, time limited, daily support, less than 4 weeks |
| R9.2 | Non-acute, non-24 hours physician cover, time limited, daily support, over 4 weeks |
| R10.1 | Non-acute, non-24 hours physician cover, time limited, lower support, less than 4 weeks |
| R10.2 | Non-acute, non-24 hours physician cover, time limited, lower support, over 4 weeks |
| R11 | Non-acute, non-24 hours physician cover, indefinite stay, 24-hours support |
| R12 | Non-acute, non-24 hours physician cover, indefinite stay, daily support |
| R13 | Non-acute, non-24 hours physician cover, indefinite stay, lower support |
| R14 | Other non-acute |
| **Day care** | |
| **DESDE-LTC code** | **Description** |
| D0.1 | Acute, Episodic acute, High intensity |
| D0.2 | Acute, Episodic acute, Other intensity |
| D1.1 | Acute, Continuous care, High intensity |
| D1.2 | Acute, Continuous care, Other intensity |
| D2.1 | Non-acute , work related, high intensity, ordinary employment |
| D2.2 | Non-acute , work related, high intensity, other work |
| D6.1 | Non-acute , work related, low intensity, ordinary employment |
| D6.2 | Non-acute , work related, low intensity, other work |
| D3.1 | Non-acute , work related, high intensity, time limited |
| D3.2 | Non-acute , work related, high intensity,time indefinite |
| D7.1 | Non-acute , work related care, low intensity, time limited |
| D7.2 | Non-acute , work related care, low intensity, time indefinite |
| D4.1 | Non-acute , non-work structured, high intensity, health related . |
| D4.2 | Non-acute , non-work structured, high intensity, education related |
| D4.3 | Non-acute , non-work structured, high intensity, social and culture related. |
| D4.4 | Non-acute , non-work structured, high intensity, other structured d |
| D8.1 | Non-acute , non-work structured, low intensity, health related |
| D8.2 | Non-acute , non-work structured, low intensity, education related |
| D8.3 | Non-acute , non-work structured, low intensity, social and culture related |
| D8.4 | Non-acute , non-work structured, low intensity, other structured day care |
| D5 | Non-acute , non structured day care, high intensity |
| D9 | Non-acute , non structured day care, low intensity |
| D10 | Other care |
| **Outpatient care** | |
| **DESDE-LTC code** | **Description** |
| O1.1 | Acute, home & mobile, 24 hours, health related care |
| O1.2 | Acute, home & mobile, 24 hours, other care |
| O2.1 | Acute, home & mobile, limited hours, health related care |
| O2.2 | Acute, home & mobile, limited hours, other care |
| O5.1.1 | No- acute, home & mobile, high intensity, health related care, 3 to 6 days a week care |
| O5.1.2 | Non-acute, home & mobile, high intensity, health related care, 7 days a week a minimum of 3 ours/day care |
| O5.1.3 | Non-acute, home & mobile, high intensity, health related care, 7 days a week |
| O5.2.1 | Non-acute , home & mobile, high intensity, other care, 3 to 6 days a week care |
| O5.2.2 | Non-acute , home & mobile, high intensity, other care, 7 days a week a minimum of 3 hours/day care |
| O5.2.3 | Non-acute , home & mobile, high intensity, other care, 7 days a week including overnight care |
| O6.1 | Non-acute , home & mobile, medium intensity, health related care |
| O6.2 | Non-acute , home & mobile, medium intensity, other care |
| O7.1 | Non acute, home & mobile, low intensity, health related care |
| O7.2 | Non acute, home & mobile, low intensity, other care |
| O8.1 | Non acute, non- mobile, high intensity, health related care |
| O8.2 | Non acute, non- mobile, high intensity, other care |
| O9.1 | Non acute, non- mobile, medium intensity, health related care |
| O9.2 | Non acute, non- mobile, medium intensity, other care |
| O10.1 | Non acute, non- mobile, low intensity, health related care |
| O10.2 | Non acute, non- mobile, low intensity, other care |

Full descriptions and examples of MTCs are provided in DESDE-LTC classification system (Salvador-Carulla et al, 2011)
